# An LC–MS Assay to Measure Superoxide Radicals and Hydrogen Peroxide in the Blood System

**DOI:** 10.3390/metabo10050175

**Published:** 2020-04-28

**Authors:** Ioannis Tsamesidis, Chinedu O. Egwu, Pierre Pério, Jean-Michel Augereau, Françoise Benoit-Vical, Karine Reybier

**Affiliations:** 1 Pharmadev, UMR 152, Université de Toulouse, IRD, UPS, 31400 Toulouse, France; chinedu.egwu@lcc-toulouse.fr (C.O.E.); pierre.perio@univ-tlse3.fr (P.P.); karine.reybier-vuattoux@univ-tlse3.fr (K.R.); 2 CNRS, LCC, Laboratoire de Chimie de Coordination, Université de Toulouse, 31077 Toulouse CEDEX 4, France; jean-michel.augereau@lcc-toulouse.fr (J.-M.A.); francoise.vical@inserm.fr (F.B.-V.); 3 Institut de Pharmacologie et de Biologie Structurale, IPBS, Université de Toulouse, CNRS, UPS, 31077 Toulouse CEDEX 4, France; 4 Alex-Ekwueme Federal University, Ndufu-Alike Ikwo P.M.B. 1010, Ebonyi State, Nigeria

**Keywords:** liquid-chromatography, mass spectrometry, superoxide radicals, hydrogen peroxide species, red blood cells, human plasma, microvesicles, *Plasmodium falciparum*

## Abstract

Red blood cells are constantly exposed to reactive species under physiological or pathological conditions or during administration of xenobiotics. Regardless of the source, its accurate quantification is paramount in the area of theragnostics, which had been elusive up until now. Even if there are a lot of approaches to evaluate the oxidative stress, very sensitive methods are missing for the blood system. We therefore sought to apply a highly sensitive approach, by liquid chromatography coupled to mass spectrometry (UPLC–MS), for the quantification of reactive species such as superoxide radical and hydrogen peroxide using dihydroethidium (DHE) and coumarin boronic acid (CBA) probes respectively through the detection of 2-hydroxyethidium (2OH-E^+^) and 7-hydroxycoumarin (COH). The use of the high-resolution mass spectrometry associated to UPLC ensured a selective detection of superoxide and hydrogen peroxide in the blood system under diverse conditions such as oxidized red blood cells (RBCs), untreated and treated parasitized RBCs. Moreover, this technique allowed the determination of reactive species in human plasma. This protocol provides a huge opportunity for in-depth study of several pathological conditions vis-a-vis their treatment in modern medicine.

## 1. Introduction

To unravel the biological roles of reactive oxygen species (ROS), the ability to detect, identify and quantify the reactive species involved at the cellular level is paramount. Oxidative stress results from an imbalance between the antioxidant system and generation of reactive species that normally takes place in healthy organism. Under conditions of oxidative stress, the increase in reactive species production leads to subsequent alteration of membrane lipids, proteins and nucleic acids [1,2]. Oxidative stress can be measured directly by the quantification of reactive oxygen, nitrogen species or indirectly by measuring the level of oxidative markers such as lipid peroxidation or antioxidant enzymes or glutathione [3]. The direct and indirect methods are usually complementary to each other in stating the oxidative state of a cell, but the direct approach is often more challenging. Several direct approaches exist for eukaryotic cells among them luminescent assays (fluorescent, chemiluminescent and bioluminescent) [4] and electron paramagnetic resonance (EPR) [5].

Fluorescent assays use certain sensors such as H_2_DCFDA (2’,7’-dichlorodihydrofluorescein diacetate) or CM-DCFDA, chloromethyl derivative of H_2_DCFDA, DHE (dihydroethidium, also called hydroethidine), CBA (B-(2-oxo-2H-1-benzopyran-7-yl)-boronic acid or coumarin boronic acid).

**Figure 1:**
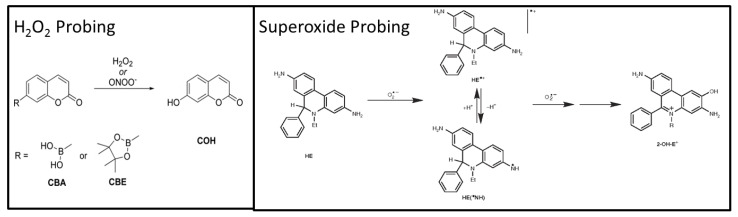
Formation of specific adducts from reaction of probes with targeted reactive oxygen species. CBA: Coumarin boronic acid (detected as the formiate adduct C_10_H_8_BO_6_^−^; *m/z* 235.0419 (th)), CBE: pinacolate ester of Coumarin boronic acid, COH: 7-hydroxycoumarin (detected as the deprotonated form C_9_H_5_0_3_^−^; *m/z* 161.0244 (th)), DHE: dihydroethidium (detected as the protonated form C_21_H_22_N_3_^+^; *m/z* 316.1808 (th)) and 2OH-E^+^: 2-hydroxyethidium (detected as a cation C_21_H_20_N_3_O^+^; *m/z* 330,1601 (th)).

However, these probes lack specificity and sensitivity for the targeted reactive species, which limit their application and subsequent interpretation of results. Indeed, the probes often form several adducts, which absorb or emit light at similar wavelengths and are also prone to redox-cycling [6]. In the same manner, the use of EPR assays has its shortfalls. Although EPR is described as the “gold standard” for the detection of radical species, the spin adducts formed after reaction of the probe with the specific radical is rapidly metabolized into the cell [7]. In these conditions, EPR analysis is suitable only for the detection of extracellular reactive species. To overcome the problem of non-selectivity, associations of fluorescent measurements with liquid chromatography (LC) were reported for eukaryotic cells for the detection of specific adducts such as 2-hydroxyethidium (2OH-E^+^) for superoxide and 7-hydroxycoumarin (COH) for hydrogen peroxide or peroxynitrite [8,9,10,11] (Figure 1). Moreover, the use of mass spectrometry coupled with LC has been proposed to unequivocally detect the desired adduct formed by the reaction of the probe with the targeted species [8,9,12,13,14,15].

In the case of red blood cells (RBCs), the options that offer the required specificity and sensitivity are limited. The need for the study of ROS in the blood system is increasingly pertinent because of several physiological (e.g., cell signaling), blood storage in transfusion units [16,17,18,19] (e.g., anaerobic and cryopreservation**)**, pathological (e.g., thalassemia and malaria) and chemotherapeutic (e.g., antimalarial, anticancer, etc.) conditions that exacerbate oxidative stress [20,21].

Until now, methods in use are especially fluorescence using H_2_DCFDA [6,22,23,24] and EPR using DMPO as a spin trap [25] for the direct quantification of reactive oxygen species in RBCs and human plasma. The shortcomings of these prevailing approaches buttress the need for a newer and more reliable approach. In this article, we report how the LC–MS method can be successfully applied to erythrocytes and human plasma for quantifying superoxide radicals and its reduced form, hydrogen peroxide in the erythrocyte system under diverse conditions.

## 2. Materials and Methods

### 2.1. Materials

DMSO, 99.9%, DHE (dihydroethidium), CBA (Coumarin boronic acid), COH (7-hydroxycoumarin), >98.0% (HPLC), phenylhydrazine, artemisinin and RPMI 1640 medium were purchased from Sigma-Aldrich, St. Quentin Fallavier, France. Formic acid (Optima for LC–MS), ammonium acetate (Optima for LC–MS), acetonitrile (HPLC gradient grade), methanol, (HPLC gradient grade) and phosphate buffer saline were purchased from Thermo Fisher Scientific, Illkirch, France. 2OH-E^+^ was synthesized following the protocol of Zielonka et al. [8].

### 2.2. Biological Materials

#### 2.2.1. Blood Sample Collection

Blood samples from healthy donors were collected in EDTA-containing tubes in the morning from Etablissement Français du Sang (EFS, Toulouse, France), responsible for ethic statements. The samples were centrifuged at 200× *g* for 5 min at 4 °C to separate the cellular components from the plasma and stored at −80 °C until the analyses were carried out.

#### 2.2.2. Cultivation of *Plasmodium falciparum*-Infected Red Blood Cells (RBCs).

Freshly drawn blood (Rh+) from healthy adults (both sexes were used) and anticoagulated with heparin was stored in citrate-phosphate-dextrose with adenine (CPDA-1) prior to use. RBCs were separated from plasma and leukocytes after centrifugation and subsequently washed three times in RPMI 1640 medium. The laboratory strains of *Plasmodium falciparum* (mycoplasma-free) were grown according to standard protocols. The parasites were maintained in RPMI 1640 medium supplemented with 5% human serum at 2% hematocrit. Both strains FcB1-Columbia and F32-Tanzania were used for developing the protocol. The *P. falciparum* parasites were maintained synchronized by treating the culture with 5% (*w/w*) D-sorbitol (1:10) at the ring stage of the erythrocytic cycle (0–12 h) as described by Lambros and Vanderberg [26]. If a very high parasitemia (≥ 80%) is required, a column chromatographic technique involving magnetic columns is used to increase the synchrony and percentage parasitemia at the trophozoite stage [27].

Before analysis the parasite culture pellet (FcB1-Columbia strain) is collected by centrifugation at 469× *g* for 5 min in a 50 mL tube. The parasitized RBCs (pRBCs) were diluted appropriately in phosphate buffer saline (PBS) to obtain 10–20 million cells/mL in 1.5 mL eppendorf tubes.

According to the study conducted, pRBCs were incubated with 200 nM ART for 1 h.

#### 2.2.3. Oxidation of Red Blood Cells

**Figure 2:**
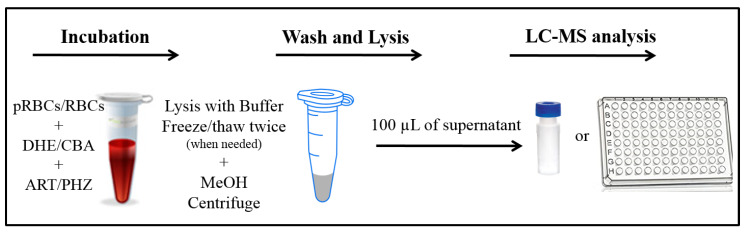
Summary of the sample the parasitized red blood cell (pRBC) or red blood cell (RBC) preparation or the LC–MS analysis. Note: pRBCs: *Plasmodium* infected red blood cells, MeOH: methanol, DHE: dihydroethydium, ART: artemisinin.

RBCs were separated from whole blood by centrifugation at 200× *g* for 5 min at 4 °C and were also washed three times in sterile phosphate buffer saline (PBS) avoiding presence of white blood cells. The RBCs (5 × 10^6^ cells) were incubated overnight (12 h) at 300 rpm at 4 °C in an Eppendorf Thermomix (Hamburg, Germany) with the ROS inducer. For the present experiment (see Figure 2 for details) RBCs were incubated with 1 mM phenylhydrazine (PHZ) in PBS. PHZ was removed the next day by washing three times with PBS. Before analysis the suspension was centrifuged at 200× *g* for 5 min at 4 °C to obtain a pellet.

#### 2.2.4. Human Plasma

Human plasma was separated from whole blood by centrifugation at 200× *g* for 5 min at 4 °C. Plasma was then transferred in a new Eppendorf while white blood cells and pellets were totally removed. The plasma was then diluted 100 times in PBS.

### 2.3. LC–MS Assays

#### 2.3.1. LC–MS Measurements

The LC–MS analysis was performed using an Ultimate 3000 UPLC system consisting of a solvent organizer SRD-3600 with a degasser, a high pressure binary gradient pump HPG-3400RS, a thermostated autosampler WPS3000TRS, an oven TCC3000SD and an UV-Visible detector DAD3000 (ThermoFisher Scientific, Courtaboeuf, France) coupled with LTQ-Orbitrap XL ETD mass spectrometer (ThermoFisher Scientific, Courtaboeuf, France).

The 2 mobile phases used for analyses consisted of A) 20 mM ammonium acetate solution prepared in water (MilliQ) pH 9 and B) acetonitrile for superoxide detection and in A) 0.1% formic acid solution prepared in water (MilliQ) and B) 0.1% formic acid prepared in acetonitrile for hydrogen peroxide detection.

For 2OH-E^+^ analysis, UPLC was performed using an UPLC Kinetex EVO C18 1.7 µm column (2.1 mm × 100 mm) at 50 °C; flow rate 400 µL/min using the following chromatographic conditions: 75% A/25% B to 95% B in 2.5 min with a non-linear gradient (concave, “curve 3”), hold for 0.5 min and then back to the initial conditions in 0.1 min. The MS analysis was performed in positive electrospray ionization (ESI) mode: spray voltage 4.2 kV; capillary temperature 300 °C, resolution 15000.

For COH analysis a UPLC Kinetex C18 1.7 µm column (2.1 mm × 100 mm) was used at 40 °C; flow rate 500 µL/min using the following chromatographic conditions: 85% A/15% B to 75% B in 2 min, to 98% B/2% A in 0.1 min. The MS analysis was performed in negative electrospray ionization (ESI) mode: spray voltage 3.2 kV and capillary temperature 300 °C, resolution 7500. The peaks of the chromatogram were integrated using Xcalibur software.

**Figure 3:**
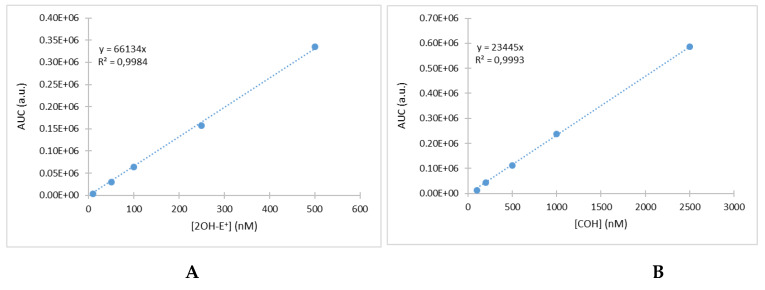
Example of the calibration curve obtained with 2OH-E^+^ (**A**) and COH (**B**).

Before each measurement, a calibration curve was prepared. The 2-hydroxyethidium (2-OH-E^+^) standard solutions were freshly prepared on the day of experiment by diluting the stock solution (50 µM) serially in the mobile phase A to obtain 6 standards from 10 to 500 nM. The COH standard solutions were also freshly prepared on the day of experiment by diluting the stock solution (10 mM) serially in aqueous methanol (50% v/v). Examples of the corresponding curves are presented in Figure 3A,B. LOD and LOQ were calculated from several calibration curves giving the following values LOD 7 nM/LOQ 24 nm for superoxide and LOD 11 nM/LOQ 37 nM for hydrogen peroxide.

#### 2.3.2. Analysis of Oxidized Red Blood Cells

Fifty microliters of the pellet were probed with 20 µM of DHE or CBA and incubated for 30 min. After centrifugation at 200× *g* for 5 min at 4 °C, the supernatant was collected for analysis. Then 100 µL of a hemolysis buffer (5 mmol/L sodium phosphate and 1 mmol/L EDTA, pH 8.0) were added to the pellet and vortexed rigorously. Before analysis, 100 µL of methanol were added to the lysed cells and to the supernatant to precipitate the cell debris. After centrifugation at 5000× *g* for 30 min at 4 °C, both samples were analyzed by the LC–MS analysis.

#### 2.3.3. Analysis of *Plasmodium Falciparum* Parasitized Red Blood Cells

A suspension of pRBCs was probed with 20 µM of DHE or CBA and incubated alongside with artemisinin (ART; 200 nM). After 1-hour incubation the suspension was centrifuged at 200× *g* for 5 min. After removing the supernatant, the pellet was washed 2–3 times in 100 µL PBS. To lyse the cells, 100 µL of the hemolysis buffer (5 mmol/L sodium phosphate and 1 mmol/L EDTA, pH 8.0) were added to the pellet placed on ice. To facilitate the lysing of the plasmodial membranes 2 freeze thaw cycles twice were performed at −80 °C and water bath respectively for about 30 min. Before analysis, 100 µL of methanol were added to the lysed cells and to the supernatant to precipitate the cell debris. After centrifugation at 5000× *g* for 30 min at 4 °C, both samples were analyzed by the LC–MS analysis.

As a control, the red blood cells were incubated with the vehicle only, i.e., using an equal amount of solvent as was used for the incubations with the chemicals of interest.

#### 2.3.4. Analysis of Human Plasma

The diluted plasma was probed with 20 µM of DHE or CBA. To separate plasma membrane vesicles (PMVs) from other components (ghost, cell fragments and debris), the suspension was centrifuged at 5000× *g* for 10 min at 4 °C. The supernatant was then collected and 100 µL of methanol was then added to lyse PMVs [28,29]. The sample was further centrifuged at 5000× *g* for 30 min at 4 °C and analyzed.

## 3. Results and Discussion

### 3.1. Detection of Reactive Oxygen Species in Oxidized RBCs

**Figure 4:**
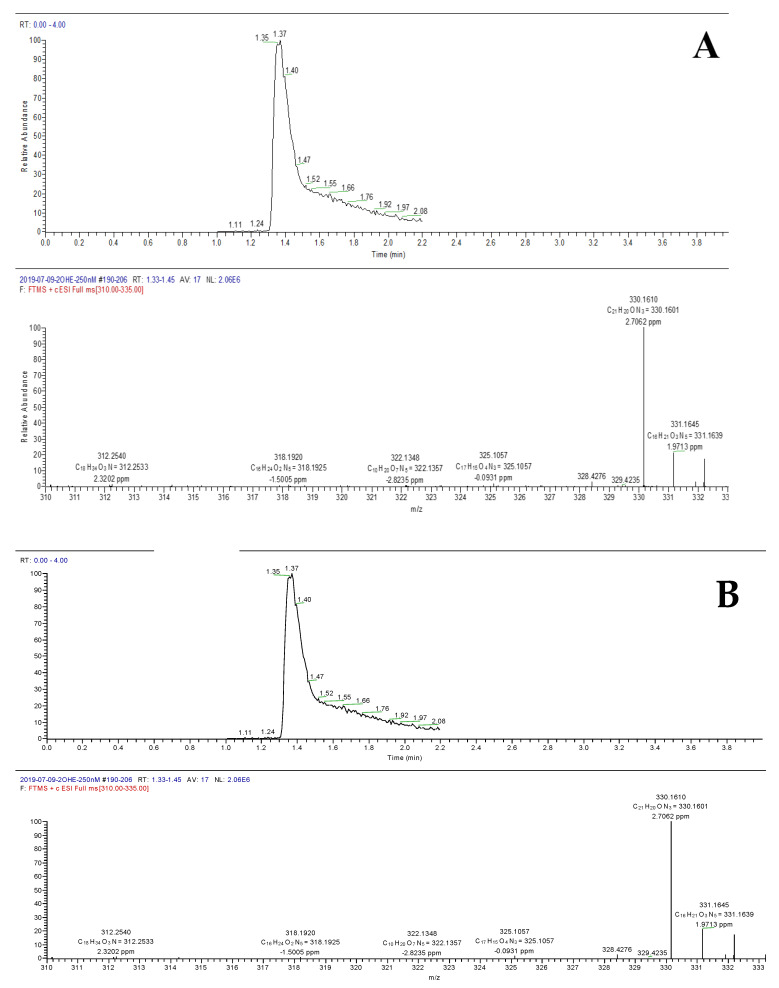
Extracted mass chromatogram based on *m/z* 330 and corresponding 2OH-E^+^ mass spectrum (**A**). Extracted mass chromatogram base on *m/z* 161 and corresponding COH mass spectrum (**B**).

**Figure 5:**
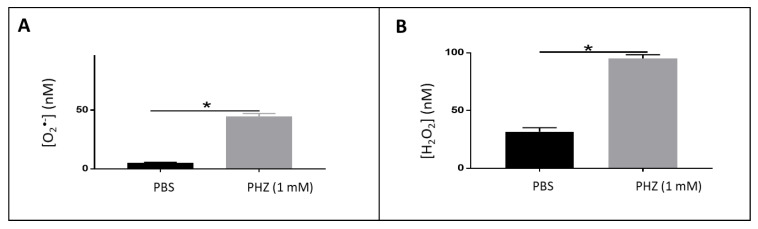
Quantification of superoxide (**A**) and H_2_O_2_ (**B**) in PHZ treated RBCs comparatively with untreated ones. Note: results represent six replicates from three independent experiments, value = mean ± SD. * = Significant (*p* < 0.05).

Treatment of RBCs with PHZ, a strong oxidant, causes selective association of oxidized alpha-globin chains with the membrane skeleton, which reduces RBC deformability, a characteristic of beta-thalassemia. Furthermore, PHZ induces deleterious oxidations in the components of erythrocytes, generating ROS and reacting with hemoglobin by changing the oxyhemoglobin into methemoglobin, hemichromes and other byproducts [30,31,32]. The nature and amount of ROS produced in such conditions were investigated using LC–MS. An example of the mass chromatogram is presented in Figure 4A,B. The corresponding concentration of superoxide and hydrogen peroxide deduced from the calibration curves are presented in Figure 5. As demonstrated, a significant increase (approximately 4 folds) of both species was observed for PHZ treated RBC in comparison with untreated ones. The concentration increased from 10 to 50 nM for O_2_^•-^ and from 25 to 100 nM for H_2_O_2_.

### 3.2. Detection of Reactive Oxygen Species in Plasmodium Falciparum Infected Erythrocytes

**Figure 6:**
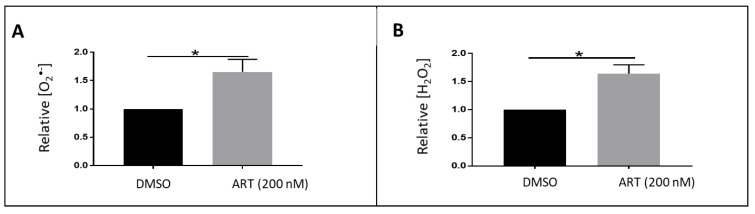
Quantification of superoxide (**A**) and H_2_O_2_ (**B**) in parasitized RBCs treated with artemisinin comparatively to the untreated ones. Note: results represents six replicates from three independent experiments, value = mean ± SD. * = Significant (*p* < 0.05).

To produce essential amino acids needed for its development, *Plasmodium falciparum* digests hemoglobin of the RBC releasing ferrous iron (Fe^2+^) species, heme that can reoxidize to form superoxide radicals and then hydrogen peroxide. Moreover, artemisinin (ART) and its derivatives act as antimalarials by inducing an overproduction of ROS after activation by heme or free iron [33]. However, the nature of the radicals produced as well as their amount has not yet been determined. Herein we applied the LC–MS to characterize the species forms both in treated and untreated pRBC. The corresponding results are presented in Figure 6. The incubation of parasitized erythrocytes for 1 h resulted in a significant increase (approximately 2 fold) in the production of O_2_^•-^ radicals and H_2_O_2_ species. It is of note that the concentration of ART used was very low (200 nM), which underlines the sensitivity of the method.

Methods allowing the specific detection and quantification of reactive species is highly needed as several clinical trials and preclinical studies [34] of new antimalarial drugs include ROS measurements to study and better understand immune response [35], disease state and progression and the health-enhancing effects of the tested antimalarials against *Plasmodium* species.

### 3.3. Detection of Reactive Oxygen Species in Human Plasma

**Figure 7:**
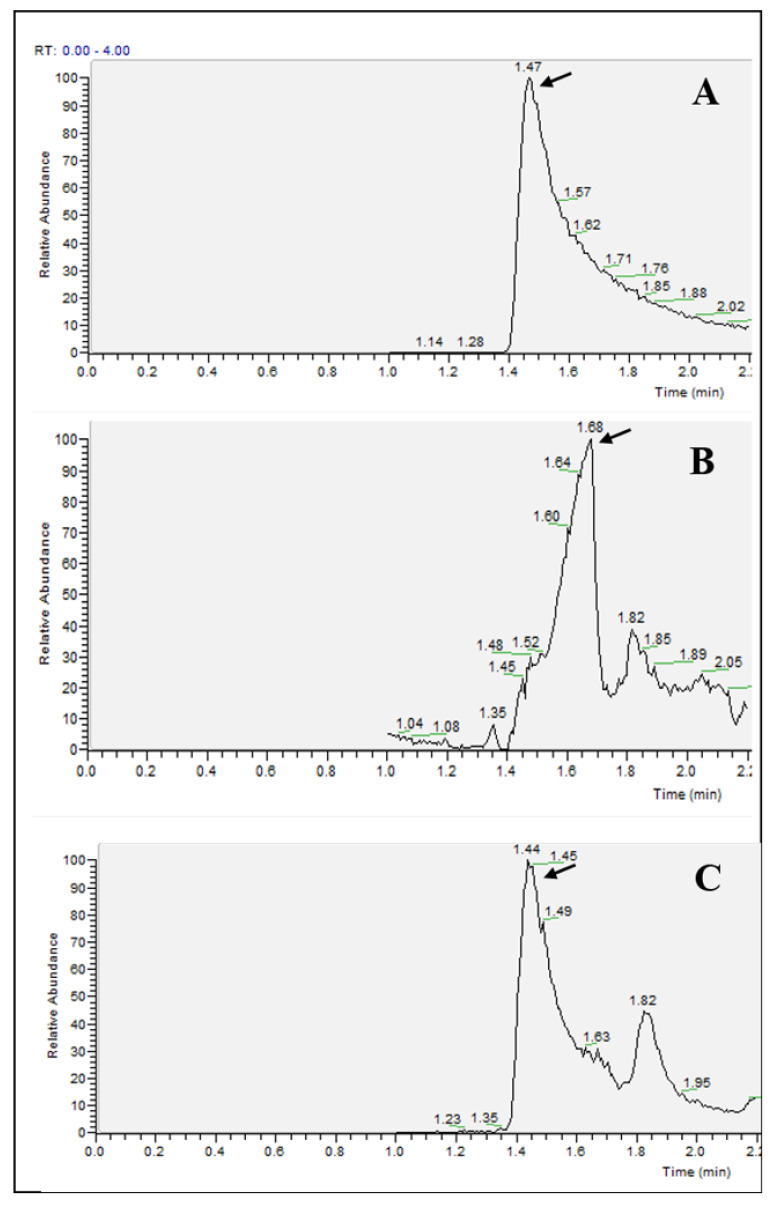
Extracted mass chromatogram base on *m/z* 330 (**A**) standard 500 nM 2OH-E^+^, (**B**) non diluted human plasma and (**C**) human plasma diluted 100 times.

Plasma membrane-vesicles (PMVs) are released into circulation because of normal and stress/pathogenic conditions. PMVs are also referred as microparticles (MPs), microvesicles (MVs) or rarely, ectosomes. They are submembrane fragments shed from the plasma membrane of red cells, platelets, white cells during cell growth, activation, proliferation, senescence and apoptosis [36]. PMVs contain a pro-oxidant or antioxidant machinery that may produce or scavenge ROS metabolic enzymes (direct effect) and can modify (activate or inhibit) the ROS content in the extra-as well as the intracellular compartments [37]. Taking into consideration that plasma contains PMVs that directly or indirectly produce or scavenge ROS, we investigated the exact amount of superoxide radicals and H_2_O_2_ species levels in the human plasma of healthy donors. It was impossible to detect ROS in non-diluted plasma as the matrix effect was observed inducing a shift of the retention time (Figure 7A,B). A 100-fold dilution of the plasma allowed us to achieve the right retention time (Figure 7C). The same phenomenon (matrix effect in non-diluted plasma) was also observed for H_2_O_2_ species detection. Moreover, a second peak was detected for a retention time of 1.82 min. This peak is attributable to a little percentage of DHE (eluted at this retention time) oxidized during ionization into 2OH-E^+.^ This second peak was not taken into account in the integration of the peak because it did not originate from superoxide.

The LC–MS allowed us to detect ROS in healthy donor’s plasma at 201 ± 13 nM for superoxide and 50 ± 9 nM for hydrogen peroxide. This result is of particular interest because it is highly desirable to measure abnormal levels of ROS in clinical samples of patients with iron overload disorders such as thalassemia and myelodysplastic syndromes [38] to better predict progression, and to improve the treatment outcomes of iron chelators.

## 4. Conclusions

The use of the high-resolution mass spectrometry associated to UPLC ensured a selective detection of superoxide and hydrogen peroxide in the blood system under diverse conditions such as oxidized RBCs, untreated and treated parasitized RBCs. Moreover, this technique allowed the determination of reactive species in human plasma. This novel method appears to be of great interest due to the increasing consideration given to ROS in various pathological states and the development of therapeutics agents.
